# Using causal frameworks to reduce bias in observational TB research: a comparison of model-building approaches

**DOI:** 10.5588/ijtldopen.26.0076

**Published:** 2026-08-11

**Authors:** L. Barcellini, S. Sauer, M. Romo, C.D. Mitnick, H. Huerga, U. Khan, C. Hewison, M.L. Rich, M.F. Franke, P.Y. Khan

**Affiliations:** 1 Department of Paediatrics, “V. Buzzi” Children’s Hospital, ASST FBF Sacco, Milan, Italy;; 2 Department of Global Health and Social Medicine, Harvard Medical School, Boston, MA, USA;; 3 Partners In Health, Boston, MA, USA;; 4 Division of Global Health Equity, Brigham and Women’s Hospital, Boston, MA, USA;; 5 Field Epidemiology Department, Epicentre, Paris, France;; 6 Interactive Research and Development (IRD) Global, Singapore, Singapore;; 7 Medical Department, Médecins Sans Frontières, Paris, France;; 8 TB Centre, London School of Hygiene & Tropical Medicine, London, UK.

**Keywords:** tuberculosis, MDR/RR-TB, HIV, observational studies, epidemiologic methods, causal inference, causal structure

## Abstract

**BACKGROUND:**

Observational studies investigating causal (aetiological) questions often address confounding bias using data-driven predictive models where variables are selected regardless of their causal role, challenging interpretation. We compared the estimated effect of HIV co-infection on end-of-treatment outcomes among people with multidrug/rifampicin-resistant TB using a causal framework model and a data-driven predictive model.

**METHODS:**

The causal framework guided confounder adjustment. Results were compared to those from models that applied alternative variable selection strategies (based on *P* values and change-in-estimate), which do not draw on domain knowledge to identify relevant variables.

**RESULTS:**

The model informed by a causal diagram indicated that people living with HIV had a 31% lower probability of achieving a successful outcome compared to those without HIV (adjusted relative risk [aRR] 0.69, 95% confidence interval [CI]: 0.41–0.98). In contrast, data-driven models produced attenuated associations (aRR 0.78, 95% CI: 0.50–1.06 and aRR 0.80, 95% CI: 0.5–1.09 for the *P* value and change-in-estimate strategies, respectively), reflecting omission of key confounders, and inappropriate inclusion of mediators and colliders.

**DISCUSSION:**

When the research question is aetiological, using a causal approach to guide variable selection ensures proper adjustment, improves interpretability, and establishes a stronger foundation for future observational research.

Observational studies may aim to describe, identify predictors, or estimate causal effects.^1^ This ambiguity can lead to confusion in the analytical approach, including variable selection and interpretation of results.^2,3^ More critically, researchers often infer causality from observational data without anchoring their analyses in a causal framework. When our aim is to determine the effect of an exposure on an outcome, we need to address confounding, a bias introduced by variables influencing both the exposure and the outcome and distorting their relationship. To reduce this bias, we select an *adjustment set* – a group of variables that we control for in the analysis – to isolate the effect of interest. Variables should be selected based on hypothesised exposure–outcome relationships, supported by existing evidence or expert knowledge. These relationships can be summarised in a causal diagram,^4^ making explicit the assumptions underpinning the analysis. This approach facilitates the identification of all confounders while avoiding adjustment for variables that could introduce bias, such as colliders (variables influenced by both the exposure and the outcome).^5^ This causal framework approach differs from prediction analyses, wherein variables are selected based on their ability to improve model performance.

We present a practical example, applying a causal framework to estimate the effect of HIV infection on treatment outcomes among individuals with multidrug-resistant/rifampicin-resistant TB (MDR/RR-TB). We compare results obtained from a model grounded in a causal framework with those from models selecting covariates without accounting for causal structure.

## METHODS

We used data from the endTB (Expand New Drug Markets for TB) observational cohort study (NCT03259269), including patients who started treatment with a bedaquiline (BDQ) and/or delamanid (DLM)–containing regimen at an endTB site across 17 countries between 1 April 2015 and 30 September 2018.^6^ Details are provided elsewhere^7^ and in the Supplementary Data.

### Outcome

MDR/RR-TB end-of-treatment outcomes were recorded using the WHO 2014 definitions.^8^ Successful treatment outcome was a composite, comprising cure and treatment completion.

### Exposure of interest

HIV status of individuals starting MDR/RR-TB treatment, established by serology or self-report prior HIV diagnosis.

### Covariates

Covariates included demographic, clinical, comorbidity, microbiological, and disease-related characteristics (detailed in the Supplementary Data).

### Analysis

We conducted multivariable logistic regression to assess the association between HIV status and successful MDR/RR-TB treatment outcome. We used marginal standardisation^9,10^ to calculate predicted probabilities and risk ratios (RRs) with 95% confidence intervals (CIs). We constructed three multivariable models to illustrate the influence of different variable selection methods on adjustment sets and effect estimates.

### Building the causal framework

**Figure 1.**
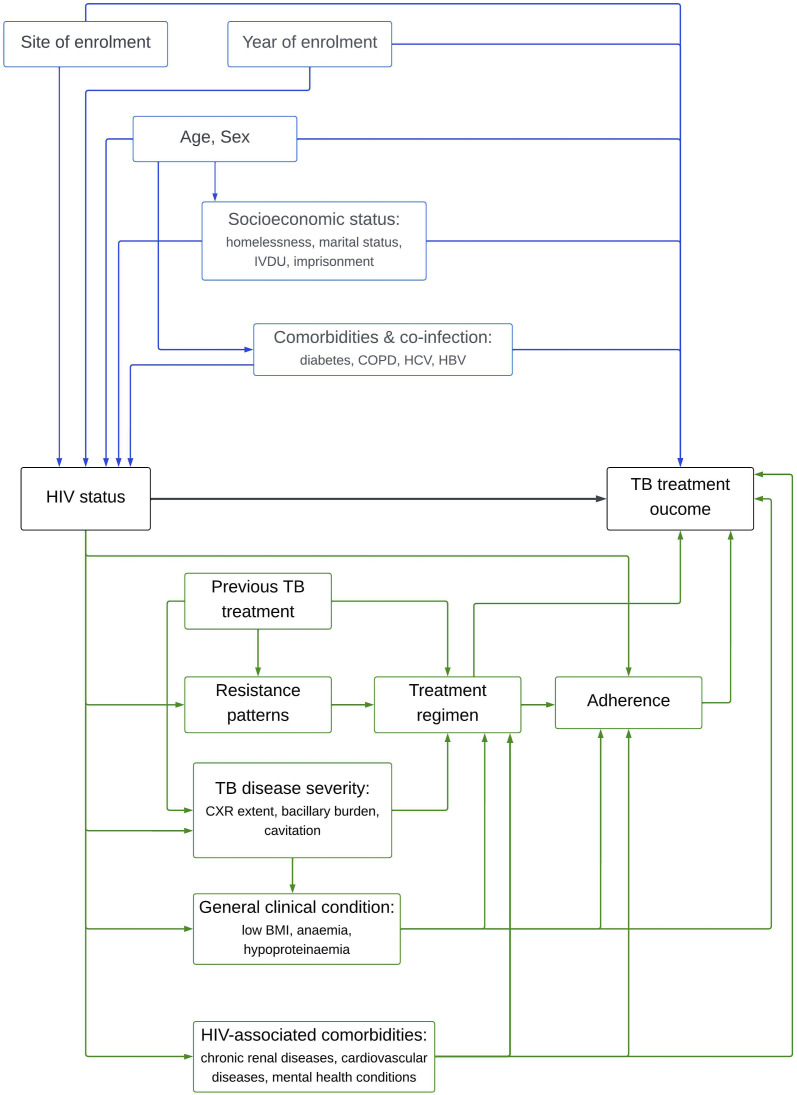
Graphical theoretical framework of the focal relationship of interest – HIV status (exposure of interest) and successful MDR/RR-TB treatment outcome (outcome of interest; composite: cured and completed). HIV status is defined without accounting for antiretroviral therapy. Variables and their measurements are represented as nodes connected by unidirectional arrows (i.e., directed), which depict hypothesised causal relationships based on prior knowledge and theory. An arrow between two nodes indicates the assumed existence and direction of a causal relationship. A path consists of one or more arrows connecting two nodes and may be open or closed; open paths transmit statistical associations, whereas closed paths do not. A causal path is defined as a sequence of arrows all pointing in the same direction from the exposure to the outcome. With respect to this focal relationship, a confounder is a common cause of both the exposure and the outcome; a mediator lies on the causal pathway between the exposure and outcome. A direct causal effect refers to the effect not operating through one or more specified mediators. The total causal effect of HIV on treatment outcome is the combined effect transmitted through all causal paths connecting the exposure and the outcome. MDR/RR-TB = multidrug-resistant/rifampicin-resistant TB; IVDU = intravenous drug use; COPD = chronic obstructive pulmonary disease; HCV = hepatitis C virus; HBV = hepatitis B virus; CXR = chest X-ray; BMI = body mass index.

To determine the appropriate adjustment set, we constructed a causal diagram (Figure 1) that synthesised existing evidence and expert reasoning to map the assumed relationships among HIV, MDR/RR-TB end-of-treatment outcome, and other variables. We identified age, sex, country, and year of enrolment as potential confounders. Age and sex influence care-seeking and treatment adherence^11^ while country and year reflect variation in care standards and delivery that may affect both diagnosis and treatment. To account for socio-economic vulnerability, we included marital status (proxy for social support), homelessness or refugee status, incarceration history, and intravenous drug use (IVDU) (proxy for social exclusion).^12,13^ We classified as confounders comorbidities that adversely influence treatment outcomes but are not downstream consequences of HIV, including diabetes, cirrhosis, chronic obstructive pulmonary disease (COPD), and chronic hepatitis B or C. Diabetes is associated with delayed sputum conversion and a higher risk of treatment failure or relapse.^14^ Cirrhosis and COPD may heighten the risk of treatment-related adverse events and restrict the use of certain anti-TB drugs.^15^ Hepatitis B and C have been linked to an elevated risk of drug-induced liver injury during TB treatment.^16^ These comorbidities frequently co-occur with HIV infection, either through biological mechanisms or shared behavioural and social risk factors. We considered drug resistance patterns to lie on the causal pathway, as more resistance can lead to poorer treatment outcomes^17^ by limiting effective therapeutic options. People living with HIV (PLHIV) may have higher levels of drug resistance due to increased TB exposure within health care settings and vulnerability to active disease (primary resistance) and to treatment-related factors such as impaired drug absorption and complex polypharmacy (secondary resistance).^18^ We considered comorbidities affected by HIV to be on the causal pathway. PLHIV are at increased risk of mental health disorders,^19^ cardiovascular and renal comorbidities due to HIV-related immune activation, and systemic inflammation, and prone to antiretroviral therapy (ART) toxicity.^20,21^ We proposed that HIV infection worsens MDR/RR-TB treatment outcomes both directly by compromising the immune response^22^ and indirectly, by delaying diagnosis and leading to greater disease severity and poorer general condition.^23^ PLHIV often present with extra-pulmonary disease, atypical pulmonary disease, or paucibacillary forms,^24^ which can hinder timely diagnosis.^25^ We considered baseline anaemia and low body mass index (BMI) as proxies for poor general condition, possibly attributable to HIV,^26,27^ placing them on the causal pathway. This assumption is debated as anaemia and low BMI can also arise from poor diet and/or food insecurity, conditions which may co-occur with but not be caused by HIV, and which lead to unfavourable outcomes via impaired cell-mediated immunity response to TB.^28^ In the latter scenario, these conditions would be confounders requiring adjustment. In the former, they are a consequence of HIV disease and may induce bias if adjusted.

### Causal model

Model 1 used a structural approach to identify confounders, selecting adjustment variables based on the causal framework. The final adjustment set included socio-demographic factors (age, sex, country, year, marital status, homelessness, refugee status, history of incarceration, IVDU, and alcohol use) and comorbid conditions that were not downstream of HIV (i.e., COPD, diabetes, hepatitis B virus [HBV], and hepatitis C virus [HCV]). We conducted sensitivity analyses considering low BMI and anaemia to occur with, rather than result from, HIV, and therefore were confounders requiring adjustment. To assess the robustness of the causal estimate to potential unmeasured confounding, we computed the E-value, which represents the minimum strength of association (on the RR scale) that an unmeasured confounder would need to have with both the exposure and the outcome, conditional on measured covariates, to fully explain away the observed effect of the primary exposure (HIV infection).^29^

### Building data-driven models

Models 2 and 3 applied data-driven strategies to variable selection. Model 2 was developed using an automated backwards stepwise selection, with a *P*-value threshold of 0.1 for variable removal and 0.05 for variable addition. Model 3 started with a parsimonious model (HIV, age, sex) and added variables incrementally based on their influence on the HIV estimate. Variables were retained in the model until changes in the estimate for HIV were less than 0.02 or the model became saturated (change-in-estimate [CIE] approach).^30^

### Missingness

We used a two-step approach to handle missing covariate data. In the causal model, we defined the adjustment set a priori based on the theoretical framework. In Models 2 (backward selection) and 3 (CIE), the missing indicator method was used during variable selection. Multiple imputation was then applied to all final models to address missingness in the selected covariates.

### Ethical statement

The endTB observational study protocol was approved by central ethics review committees for each consortium partner, and local ethics approval was obtained in all endTB countries. Participants provided written informed consent for inclusion in the observational cohort.

## RESULTS

The analysis sample included 2,220 individuals with MDR/RR-TB (Supplementary Data Figure S1), of whom 262 (11.8%) were PLHIV (Supplementary Data Table S1). Participants with missing HIV status (n = 1) or missing treatment outcome (n = 92) were excluded from the analysis sample (Supplementary Data Figure S1). Among PLHIV, 198 were on ART (198/262, 75.6%). Overall, 64.1% (168/262) of PLHIV experienced a successful treatment outcome compared to 79.8% (1,555/1,958) of HIV-negative individuals (Supplementary Data Table S2). Missingness across key variables is reported in Supplementary Data Table S3.

### Comparison of modelling approaches

**Figure 2.**
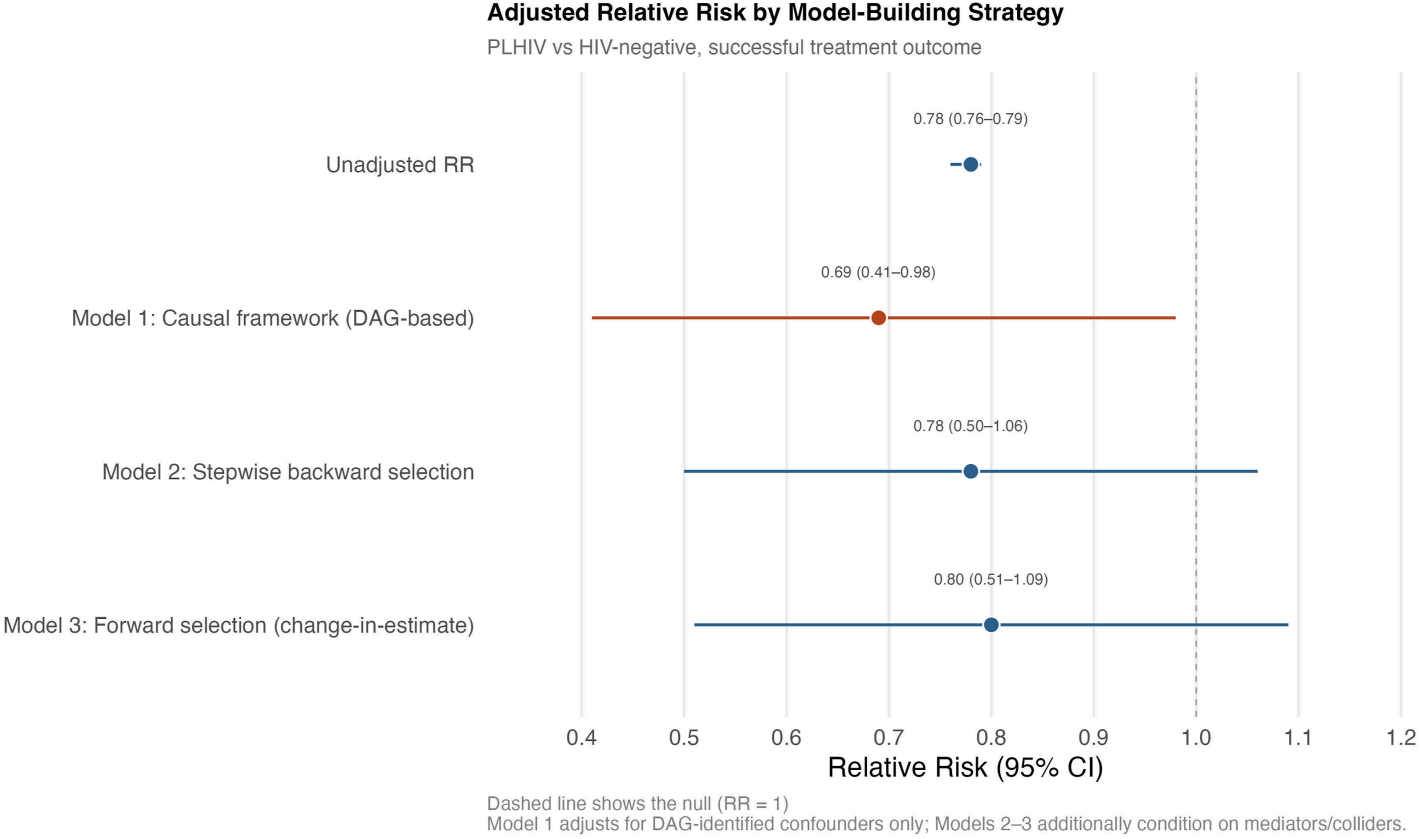
Forest plot of adjusted relative risk of successful MDR/RR-TB treatment outcome among people living with HIV compared with HIV-negative patients, by model-building strategy. MDR/RR-TB = multidrug-resistant/rifampicin-resistant TB; PLHIV = people living with HIV; CI = confidence interval.

Adjustment sets and effect estimates are shown in Table 1 and Figure 2.

**Table 1.** Adjustment set, predicted probabilities, and relative risk (RR) of the effect of HIV status on multidrug-resistant/rifampicin-resistant TB treatment outcomes using different model-building strategies.

Model-building strategy	Adjustment set	Analysis sample	Predicted probability of successful outcome (95% CI)	Adjusted RR	95% CI
Unadjusted RR	–	2,220		0.78	0.76–0.79
Model 1: causal framework	Age, sex, site, year, history of incarceration, marital status, IVDU, alcohol use, homelessness, COPD, diabetes, HCV, HBV	2,217^A^	HIV-negative: 0.79 (0.77–0.80)	0.69	0.41–0.98
			PLHIV: 0.71 (0.64–0.78)		
Model 2: stepwise backwards selection	Age, sex, site, chronic renal disease, diabetes, bilateral lung involvement, depression, marital status, history of incarceration, homelessness, positive baseline culture, psychiatric disorders, HCV, HBV, low BMI	2,217^A^	HIV-negative: 0.78 (0.77–0.82)	0.78	0.50–1.06
			PLHIV: 0.73 (0.67–0.80)		
Model 3: forward selection using change-in-estimate	Age, sex, anaemia, extensive disease, HCV, site, HBV, IVDU, low BMI, chronic renal disease, year	2,217^A^	HIV-negative: 0.78 (0.76–0.80)	0.80	0.51–1.09
			PLHIV: 0.74 (0.67–0.80)		

CI = confidence interval; IVDU = intravenous drug use; COPD = chronic obstructive pulmonary disease; HCV = hepatitis C virus; HBV = hepatitis B virus; PLHIV = people living with HIV; BMI = body mass index.

A Analysis sample n = 2,220, dropped Kenya (3 observations).

### Model 1: Causal model

**Figure 3.**
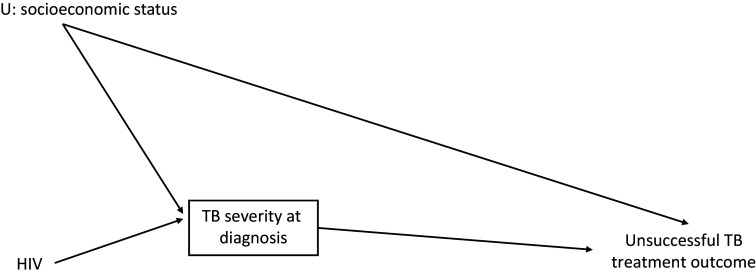
Causal diagram illustrating collider bias due to conditioning on TB severity. TB disease severity is influenced by both HIV status and other factors and, in turn, affects study inclusion and treatment outcomes. TB severity therefore lies on a collider path between HIV status and treatment outcome. A collider path is a closed path between an exposure and an outcome that passes through a variable receiving two or more arrows and, by default, does not transmit statistical associations. Conditioning on TB severity (indicated by the square around the node) opens this path, creating a non-causal backdoor path between HIV and treatment outcome through severity, and can induce a spurious association between exposure and outcome, even in the absence of a true causal effect.

Confounders were selected based on the theoretical causal framework described above. The adjusted relative risk (aRR) for the effect estimate of HIV on successful treatment outcome was 0.69 (95% CI: 0.41–0.98). In a sensitivity analysis additionally adjusting for BMI and anaemia, the aRR was 0.73 (95% CI: 0.47–1.13) (Supplementary Data Table S4). The calculated E-value for the point estimate was 2.26 (Figure 3).

### Model 2: Backward selection

Model 2 included a larger number of covariates such as extensive disease (high smear grade with cavitary disease), anaemia, chronic renal disease, depression, psychiatric disorders, and low BMI. When confronted with the causal framework used to build model 1, extensive disease acts as a collider, while the other variables are mediators on the causal pathway between HIV and successful treatment outcomes (Table 2). The aRR for HIV from model 2 was 0.78 (95% CI: 0.50–1.06).

**Table 2.** Comparison of variable selection strategies across models and implications for bias. Confounders, mediators, and colliders are based on the proposed causal framework.

Model	Variable selection approach	Types of variables included	Rationale/notes
Model 1	Theory-driven (causal framework)	Confounders only – all identified confounders included based on theoretical reasoning^A^	Based on theoretical framework; aimed at estimating the total causal effect of HIV on MDR/RR-TB treatment outcome
Model 2	Stepwise backwards selection (*P* value based)	Mix of confounders, mediators (low BMI), and colliders (e.g., ‘extensive disease’ – see TB severity causal model in Figure 2)	Variable selection driven by statistical significance; ignores causal structure and may introduce bias by including mediators or colliders and omitting key confounders
		Confounders omitted: IVDU, alcohol use, homelessness, COPD, diabetes	
Model 3	Change-in-estimate (forward selection)	Mix of confounders, mediators (anaemia, low BMI), and colliders (e.g., ‘extensive disease’ – see TB severity causal model in Figure 2)	Variable selection driven by statistical significance; ignores causal structure and may introduce bias by including mediators or colliders and omitting key confounders
		Confounders left out of model (history of incarceration, marital status, homelessness, COPD, diabetes)	

MDR/RR-TB = multidrug-resistant/rifampicin-resistant TB; BMI = body mass index; IVDU = intravenous drug use; COPD = chronic obstructive pulmonary disease.

A Under a structural definition of confounders, a sufficient adjustment set for a specific *estimand* (i.e., the causal effects being sought to determine) refers to a set of variables that, when fully controlled for, leads to an unbiased estimate by closing non-causal paths and keeping causal paths open (Hernán MA, et al. Causal Knowledge as a Prerequisite for Confounding Evaluation: An Application to Birth Defects Epidemiology. American Journal of Epidemiology. 2002; 155(2):176-184).

### Model 3: CIE approach

In Model 3, the adjustment set included age, sex, site, year, anaemia, chronic renal disease, IVDU, low BMI, HBV, HCV, and extensive disease. This resulted in the inclusion of colliders (extensive disease) and mediators (anaemia, low BMI, chronic renal disease), while omitting confounders (Table 2). This model produced an aRR of 0.80 (95% CI: 0.51–1.09) for HIV.

## DISCUSSION

This analysis illustrates the application of a causal framework to address an aetiological question, namely estimating the total effect of HIV on MDR/RR-TB treatment outcomes. Using causal theory to inform selection of adjustment variables, we found that PLHIV had a 31% lower probability of achieving a successful treatment outcome compared to those without HIV (aRR 0.69, 95% CI: 0.41–0.98). In contrast, the data-driven models estimated lower probabilities of 22% (aRR 0.78, 95% CI: 0.50–1.06) and 20% (aRR 0.80, 95% CI: 0.51–1.09). This attenuation is consistent with inappropriate adjustment for variables that lie on the causal pathway between HIV and treatment outcome. By conditioning on mediators, these models block part of the total causal effect of HIV on treatment outcomes, biasing the estimate toward the null.

This finding underscores the importance of selecting adjustment variables based on causal reasoning rather than statistical associations alone. Data-driven selection strategies may exclude important confounders or adjust for mediators or colliders, introducing bias and obscuring the true relationship between the exposure and outcome. Variables that distort causal inference may remain strong predictors of the outcome, which explains their persistence in data-driven models. This illustrates the ambiguity inherent in many observational studies, where variables informative for description or prediction may be inappropriate for estimating causal effects, underscoring the need to explicitly define the analytical objective. This is also true for non-data-driven approaches, such as simply including known ‘risk factors’ for the outcome in the adjustment set. This latter approach requires further vetting of how the risk factors relate to the exposure and outcome of interest to avoid the same pitfalls of data-driven selection strategies.

In practice, failure to encode causal structure can bias estimates through three main mechanisms: 1) Omission of confounders: Variable selection based solely on *P* values may overlook confounders that have weak statistical associations with the outcome in the sample but strong theoretical links to both exposure and outcome.^31^ In our analysis, IVDU was excluded from the data-driven model despite being a known confounder. 2) Inclusion of mediators: Failing to consider the causal structure may lead to adjustment for mediators. In model 3, the adjustment set includes low BMI and anaemia, both of which are likely mediators. Because mediators lie along the causal pathway through which the exposure exerts its effect, adjusting for them blocks part of that pathway and attenuates the estimated association, leading to an underestimation of the total effect.^32^ 3) Inclusion of colliders*:* Collider stratification bias is a type of selection bias that occurs when we adjust for a variable influenced by both the exposure and outcome. This can occur at both design and analysis stages, through restricting the study population or statistical adjustment.^33^ Both data-driven models adjusted for proxies of poor medical condition, a likely collider. HIV worsens medical conditions at diagnosis due to diagnostic delays and immune dysfunction, while socio-economic factors may influence both condition and MDR/RR-TB treatment outcomes. Adjusting for poor condition can therefore induce spurious associations and bias the estimated effect (Figure 3).

This illustrative example shows how causal frameworks guide analysis and interpretation; further refinements could strengthen the estimation of the causal effect of HIV on MDR/RR-TB outcomes. Causal model assumptions: Placing certain variables within the causal framework is debatable and inherently complex – for instance, the role of BMI and anaemia. We conducted sensitivity analyses in which low BMI and anaemia were treated as upstream risk factors that co-occur with HIV rather than as downstream consequences, resulting similar estimates. Importantly, ART was not accounted for in this analysis. The positions of several variables within the causal framework would likely differ between people on ART with viral suppression and those not receiving ART. Addressing this would constitute a different causal question and would require explicitly accounting for these differences. Selection bias: TB disease severity influences both study inclusion and treatment outcomes (Figure 2). Because the underlying cohort study was limited to microbiologically confirmed MDR/RR-TB, PLHIV with pulmonary disease were more likely to be included, whilst those with paucibacillary or atypical presentations, which is nearly half of HIV-associated TB,^34^ may have been missed. If present, this selection bias could lead to an underrepresentation of advanced HIV-associated MDR/RR-TB and an over-estimation of treatment success among PLHIV, resulting in upward bias in the HIV effect estimate. In our cohort, this limitation common to most MDR/RR-TB programmatic cohorts remains; the use of both Xpert and culture-based inclusion criteria was intended to mitigate this source of selection bias. Residual confounding: This was a secondary analysis of data originally collected to evaluate the safety and effectiveness of BDQ and DLM–containing regimens. Not all potential confounders of the HIV and MDR/RR-TB treatment outcome relationship were recorded. Unmeasured factors that influence both HIV and treatment success could bias the estimates. We computed the E-value for the causal model estimate. The E-value of 2.26 for the point estimate suggests that a moderately strong unmeasured confounder would be required to fully explain away the observed effect. Finally, some argue that causal questions should focus on modifiable exposures.^35^ In this analysis, HIV status is defined at the point of MDR/RR-TB treatment initiation, and the causal effect we are trying to estimate can be framed as the effect of initiating MDR/RR-TB treatment whilst living with HIV versus initiating treatment without HIV. While HIV status itself is not directly modifiable in the absence of cure, key determinants along the HIV care pathway are, including prevention of HIV acquisition and provision of effective ART to ameliorate immune dysfunction. For these reasons, causal questions about HIV therefore remain valuable: by quantifying its effects on specific health outcomes, it can inform and strengthen the case for investment in preventive and therapeutic interventions. Future studies with more granular data, such as CD4 cell counts, HIV viral load, and ART history, would enable a more nuanced evaluation of how variations in immune status, stage of HIV disease, and viral control modify MDR/RR-TB treatment outcomes.

## CONCLUSION

This study demonstrates the practical application of a causal framework to estimate the effect of HIV status on MDR/RR-TB treatment outcome. It shows how variable selection strategies grounded in causal reasoning can alter adjustment sets and effect estimates, highlighting the value of explicit causal thinking in strengthening the credibility and interpretability of epidemiological analyses.

## Supplementary Material
