# Supplementary material for: Using causal frameworks to reduce bias in observational TB research: a comparison of model-building approaches
Source: IJTLD Open. 2026 Aug 11;3(8):494–501. doi: 10.5588/ijtldopen.26.0076 (PMC13460431; doi:10.5588/ijtldopen.26.0076)
Supplement: Supplementary file 1 [file ijtldopen26-0076_supplementarydata1.pdf]

Supplementary material

Material and Methods

Description of the End TB Observational Cohort

Study Setting

From April 2015 to September 2018, enrolment for this study took place in 17 different countries. These countries were chosen to represent the diverse settings where MDR-TB is a significant issue. This included countries with high and low levels of second-line drug resistance, a mix of public and private health providers, high and low HIV prevalence, inpatient and outpatient management (1). The countries included were Armenia, Bangladesh, Belarus, Democratic People's Republic of Korea, Ethiopia, Georgia, Haiti, Indonesia, Kazakhstan, Kenya, Kyrgyzstan, Lesotho, Myanmar, Pakistan, Peru, South Africa, and Vietnam(1).

Data source

A standardised study protocol was employed for data collection across all sites (1). The dataset for this analysis contained anonymised data on patient characteristics collected before initiating BDQ and/or DLM treatment (baseline) and treatment outcome. The dataset exclusively comprised patients from sites with HIV testing, excluding those from the Democratic People's Republic of Korea due to no HIV testing at their site.

Exposure of interest

The primary exposure in this analysis is HIV status at baseline. HIV testing was offered to all participants at enrolment. Testing procedures and management followed National guidelines. Information about ART at the study-drugs start was also collected.

Outcome measure and definition

Post-treatment outcomes were recorded by clinicians on a standardised form at scheduled visits at the end of treatment. MDR-TB treatment outcomes were recorded based on the WHO criteria (2014) and classified as follows(1, 2):

- Treatment Failed: Treatment terminated or need for regimen change of at least two anti-TB drugs because of:
    - lack of conversion by the end of the intensive phase,
    - or bacteriological reversion in the continuation phase,
    - or evidence of additional acquired resistance to fluoroquinolones or injectable drugs,
    - or adverse drug reactions.
- Loss to Follow-Up (LTFU): Patients who did not start treatment or whose treatment was interrupted for two consecutive months or more.
- Died: A patient who dies for any reason during treatment.
- Cured: Treatment completed as recommended by the national policy without evidence of failure AND three or more consecutive cultures taken at least 30 days apart are negative after the intensive phase.
- Treatment Completed: Treatment completed as recommended by the national policy without evidence of failure BUT no record that three consecutive cultures taken at least 30 days apart are negative after the intensive phase.

Unsuccessful treatment outcome was defined as the sum of treatment failures, LTFU, and deaths (composite outcome).

Covariates definition

At the start of treatment, patients underwent a full medical history, a clinical examination, Chest X-Ray, ECG, laboratory blood tests sputum smear microscopy, culture and drug-susceptibility testing (DST)(1).

The information included in the dataset is the following:

- Sociodemographic information: collected at the baseline consultation included age, gender, site and year of enrolment, intravenous drug use within the past year, history of incarceration, homelessness or refugee status, and marital status.
- Treatment regimen:
    - Number of drugs in the initial regimen. This was a categorical variable ranging from 0 to 10.
    - Number of effective drugs in the initial regimen. The likely effectiveness was determined based on two criteria: (1) if all testing (phenotypic or genotypic) confirmed susceptibility to- the drug, or (2) if no resistance to the drug was reported and the patient had not taken the drug for one month or more. This information was recorded as a categorical variable ranging from 1 to 8.
- Microbiological characteristics:
    - Smear microscopy. Sputum samples for smear microscopy were obtained from samples collected closest to the start of a BDQ and/or DLM-containing regimen (i.e., baseline) and within 90 days before it. In this dataset, information on smear microscopy was recorded as follows:
        - A binary variable indicating the smear microscopy result, categorised as either "positive" or "negative."
        - An ordinal categorical variable representing different smear grades, including "negative smear," "scanty 1-3 AFB (acid fast bacilli)," "scanty 4-9 AFB," "1+ AFB," "2+ AFB," and "3+ AFB."
    - Culture: a binary variable was used to register culture as positive or negative. A positive baseline sputum culture was defined as a positive culture obtained from the sputum sample collected closest to the baseline and within 90 days prior to it.
    - The resistance patterns were assessed through either phenotypic or genotypic testing. They were categorised as "RR/MDRTB: without additional resistance to FQ/injectable drugs", "RR/MDRTB: without testing to FQ and injectable", "RR/MDRTB + injectable resistance", "RR/MDRTB + FQ resistance", and "RR/MDRTB + both FQ and Injectable resistant".
- Disease Severity:
    - Cavitary disease: cavitations were identified by baseline chest X-rays and recorded as a binary.
    - The extent of pulmonary TB disease. The TB disease's extent was categorised as either unilateral or bilateral based on the initial Chest X-ray.
    - TB treatment history was classified into four categories: "no previous treatment", "previous treatment with first-line drugs", "previously treated with second-line drugs", and "history unclear".
    - Patient's General Condition:
        - Malnourishment: The patient's Body Mass Index (BMI) was measured before the treatment. The dataset included a binary variable to indicate if the patient's BMI was less than 18.5 kg/m^2.
        - Haemoglobin Level (g/dL) at baseline, recorded as a continuous variable
- Comorbidities:
    - Diabetes mellitus (DM): DM was defined as random or fasting blood sugar > 200 mg/dL or HbA1c result > 6·5% and registered as a binary variable.

    - Presence of cardiovascular disease, cirrhosis, cancer, chronic kidney disease, mental illnesses (depression and other psychiatric conditions) and chronic obstructive pulmonary disease (COPD) based on past medical history collected at the initial consultation. All these variables were coded as binary.
- Co-infections:
  Upon enrolment, all participants were offered testing for hepatitis B and hepatitis C. The primary test for screening for chronic active HBV was the HBV surface antigen (HBsAg) test. A positive hepatitis C antibody through either ELISA or point-of-care rapid testing, or a polymerase chain reaction (PCR)/genotype test, was considered evidence of HCV infection. Results were recorded as either positive or negative.

Figure S1. Study flowchart

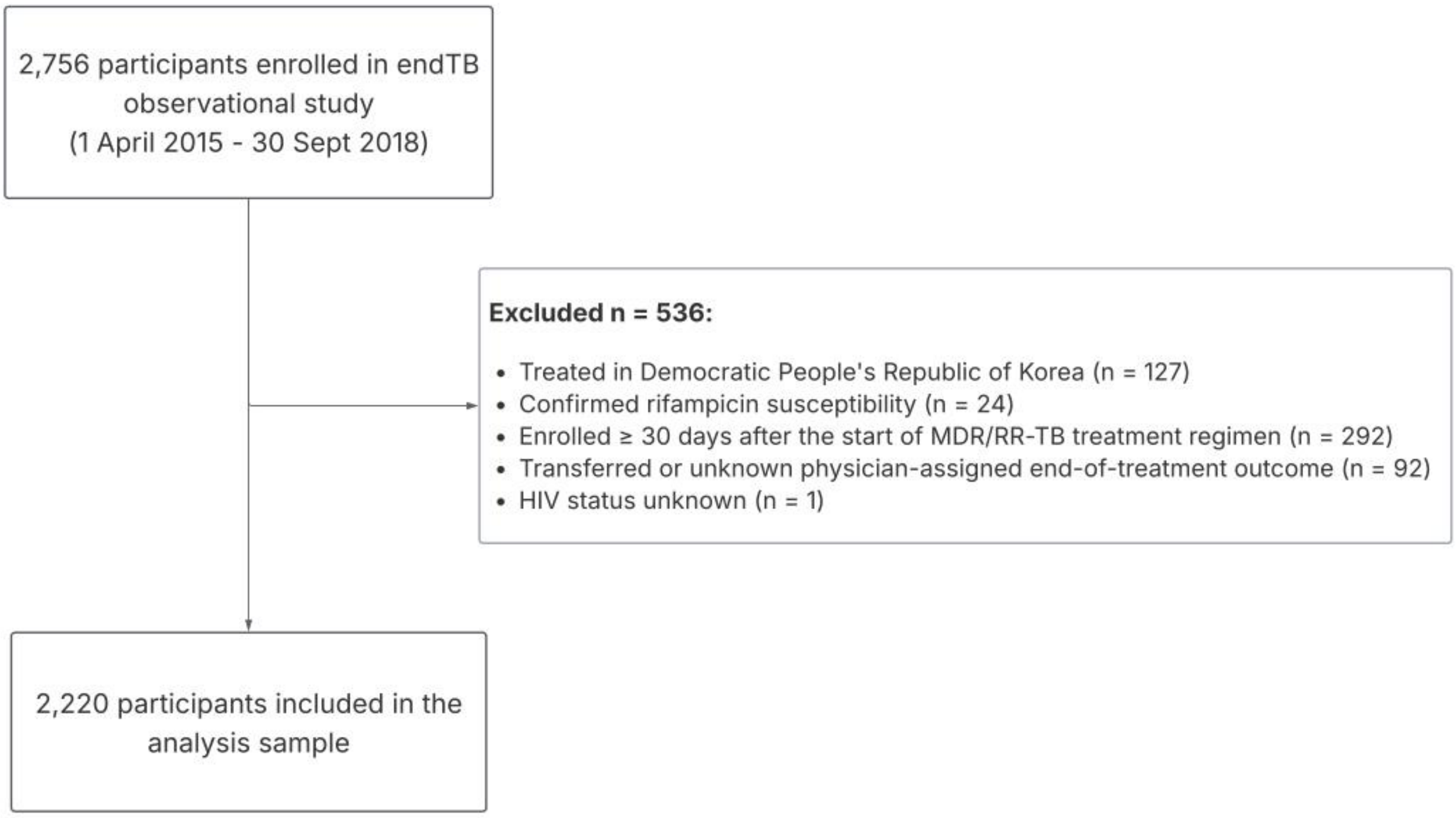

Table S1. Characteristics of the study participants overall and stratified by HIV status

	Total N=2,220	HIV-negative N=1,958 (88.2%)	HIV-positive N=262 (11.8%)
**Age in years, median (IQR)**	35 (27-47)	35 (26-46)	39 (31-47)
**Gender**			
Male	1,428 (64.3%)	1,259 (64.3%)	169 (64.5%)
Female	792 (35.7%)	699 (35.7%)	93 (35.5%)
**Baseline resistance profile**			
MDR/RR-TB: without resistance FQ /inj	385 (17.3%)	322 (16.5%)	63 (24.0%)
MDR/RR-TB: without testing FQ and inj	218 (9.8%)	135 (6.9%)	83 (31.7%)
MDR/RR-TB + inj resistance	325 (14.6%)	294 (15.0%)	31 (11.8%)
MDR/RR-TB + FQ resistance	576 (26.0%)	537 (27.4%)	39 (14.9%)
MDR/RR-TB + FQ and inj resistant	716 (32.3%)	670 (34.2%)	46 (17.6%)
**Smear at baseline***			
Negative	982 (48.5%)	865 (47.2%)	117 (61.9%)
Positive	1,041 (51.5%)	969 (52.8%)	72 (38.1%)
**Culture at baseline***			
Negative	696 (35.0%)	653 (36.1%)	43 (24.4%)
Positive	1,291 (65.0%)	1,158 (63.9%)	133 (75.6%)
**Previous TB treatment***			
No previous treatment	234 (10.5%)	161 (8.2%)	73 (28.1%)
Treated with 1st line drugs	316 (14.3%)	259 (13.2%)	57 (21.9%)
Treated with 2nd line drugs	1,668 (75.2%)	1,538 (78.6%)	130 (50.0%)
**Bilateral lung involvement***			
No	651 (33.1%)	604 (33.8%)	47 (25.5%)
Yes	1,319 (66.9%)	1,182 (66.2%)	137 (74.5%)
**Extensive disease (cavitation and smear-positive ≥ 3+)***			
No	1,839 (82.8%)	1,599 (81.7%)	240 (91.6%)
Yes	381 (17.2%)	359 (18.3%)	22 (8.4%)
**Malnutrition (BMI <18.5 kg/m^2) ***			
No	1,325 (60.5%)	1,203 (61.9%)	122 (49.8%)
Yes	864 (39.5%)	741 (38.1%)	123 (50.2%)
**Haemoglobin (g/dl) ***			
≤ 9.9	266 (11.9%)	175 (8.9%)	91 (34.7%)
10-12.9	1,389 (62.3%)	1,258 (63.9%)	131 (50.0%)
≥13	503 (22.5%)	479 (24.3%)	24 (9.2%)
**At least 5 effective drugs in baseline regimen**			
No	1,082 (48.7%)	983 (50.2%)	99 (37.8%)
Yes	1,138 (51.3%)	975 (49.8%)	163 (62.2%)
**Chronic renal insufficiency***			
No	2,067 (97.7%)	1,887 (98.1%)	191 (94.1%)
Yes	49 (2.3%)	37 (1.9%)	12 (5.9%)
**COPD***			

No	2,071 (97.9%)	1,866 (98.1%)	205 (96.2%)
Yes	45 (2.1%)	37 (1.9%)	8 (3.8%)
**Heart disease***			
No	2,039 (96.3%)	1,835 (96.3%)	204 (96.2%)
Yes	79 (3.7%)	71 (3.7%)	8 (3.8%)
**Depression***			
No	2,036 (96.6%)	1,846 (97.7%)	190 (96.0%)
Yes	51 (2.4%)	43 (2.3%)	8 (4.0%)
**Psychiatric disease***			
No	2,050 (98.0%)	1,863 (98.4%)	187 (94.9%)
Yes	41 (2.0%)	31 (1.6%)	11 (5.1%)
**Diabetes***			
No	1,849 (84.6%)	1,631 (83.6%)	218 (92.8%)
Yes	336 (15.4%)	319 (16.4%)	17 (7.2%)
**Hepatitis C infection***			
No	1,952 (88.5%)	1,741 (89.1%)	211 (83.7%)
Yes	253 (11.5%)	211 (10.9%)	41 (16.3%)
**Hepatitis B infection***			
No	2,106 (95.6%)	1,876 (96.2%)	230 (91.3%)
Yes	96 (4.4%)	74 (3.8%)	22 (8.7%)
**Married***			
No	1,013 (45.9%)	878 (45.0%)	135 (52.7%)
Yes	1,193 (54.1%)	1,072 (55.0%)	121 (47.3%)
**Homeless/refugee status***			
No	2,001 (93.8%)	1,766 (93.7%)	235 (94.0%)
Yes	133 (6.2%)	118 (6.3%)	15 (6.0%)
**IVDU in past year***			
No	2,090 (97.7%)	1,868 (98.4%)	222 (92.5%)
Yes	49 (2.3%)	31 (1.6%)	18 (7.5%)
**History of incarceration***			
No	1,922 (87.3%)	1,696 (87.2%)	226 (87.9%)
Yes	280 (12.7%)	249 (12.8%)	31 (12.1%)
**History of alcohol use***			
No	1898 (87.5%)	1703 (88.8%)	195 (78.0%)
Yes	270 (12.5%)	215 (11.2%)	55 (22.0%)
**Year**			
2015	139 (6.3%)	124 (6.3%)	15 (5.7%)
2016	623 (28.0%)	567 (29.0%)	56 (21.4%)
2017	715 (32.2%)	624 (31.9%)	91 (34.7%)
2018	743 (33.5%)	643 (32.8%)	100 (38.2%)

FQ = fluroquinolone; inj = second-line injectable; COPD = chronic obstructive pulmonary disease; IVDU = intravenous drugs users
*Missing values (shown in Table S5)

Table S2: Distribution of MDR/RR-TB treatment outcomes by HIV status

	Total N = 2,220 (col %)	HIV-negative N = 1,958 (col %)	HIV-positive N = 262 (col %)
**Physician-assigned outcome**			
Cured	1,615 (72.8%)	1,456 (74.4%)	159 (60.7%)
Completed	108 (4.9%)	99 (5.1%)	9 (3.4%)
Died	213 (9.6%)	150 (7.7%)	63 (24.1%)
Failed	101 (4.6%)	95 (4.9%)	6 (2.3%)
Loss to follow up	183 (8.3%)	158 (8.1%)	25 (9.5%)
**Successful treatment outcome**			
Yes	1,723 (77.6%)	1,555 (79.8%)	168 (64.1%)
No	497 (22.4%)	405 (20.6%)	94 (35.9%)

Table S3*:* Missing data overall and stratified by HIV status.

Variable	Missing data n (%), N=2,220	HIV negative N=1958	HIV positive N=262
**Smear at baseline**	197 (8.87)	124 (6.33)	73 (27.86)
**Culture at baseline**	233 (10.50)	147 (7.51)	86 (32.82)
**Alcohol use**	52 (2.34)	40 (2.04)	12 (4.58)
**Prison**	18 (0.81)	13 (0.66)	5 (1.91)
**IVDU**	81 (3.65)	59 (3.01)	22 (8.40)
**Homelessness**	86 (3.87)	74(3.78)	12 (4.58)
**Marital status**	14 (0.63)	8(0.41)	6 (2.29)
**Chronic renal insufficiency**	104 (4.68)	45(2.30)	59 (22.52)
**Chronic obstructive pulmonary disease**	104 (4.68)	55 (2.81)	49 (18.70)
**Heart disease**	102 (4.59)	52 (2.66)	50 (19.08)
**Depression**	133 (5.99)	69 (3.52)	64 (24.43)
**Other psychiatric disease**	129 (5.81)	64 (3.27)	65 (24.81)
**Bilateral lung involvement**	250 (11.26)	172(8.78)	78 (29.77)
**HCV**	15 (0.68)	5 (0.26)	10 (3.82)
**HBV**	18 (0.81)	8 (0.41)	10 (3.82)
**Haemoglobin**	32 (1.44)	18 (0.92)	14 (5.34)
**Baseline diabetes**	35 (1.58)	8(0.41)	27 (10.31)
**Malnutrition (BMI <18.5)**	31 (1.40)	14 (0.72)	17 (6.49)

Table S4: Sensitivity analyses exploring alternative causal assumptions for BMI and anaemia. The primary model reflects the prespecified causal framework for the effect of HIV on successful MDR/RR-TB outcome, while sensitivity analyses treat low BMI and/or anaemia as baseline confounders rather than downstream consequences of HIV. Estimates are shown for comparison with the main model.

Model-building strategy	Adjustment set	Analysis sample	Adjusted relative risk	95% CI
Unadjusted RR	-	2,220	0.78	0.76 – 0.79
Model 1: causal framework	Age, sex, site, year, history of incarceration, marital status, IVDU, alcohol use, homelessness, COPD, diabetes, HCV, HBV	2,217§	0.69	0.41 – 0.98
Sensitivity analysis adjusting for low BMI and anaemia	Age, sex, site, year, history of incarceration, marital status, IVDU, alcohol use, homelessness, COPD, diabetes, HCV, HBV, low BMI, anaemia	2,217§	0.73	0.47- 1.13
Sensitivity analysis adjusting for low BMI only	Age, sex, site, year, history of incarceration, marital status, IVDU, alcohol use, homelessness, COPD, diabetes, HCV, HBV Low BMI	2,217	0.73	0.47- 1.12
Sensitivity analysis adjusting for anaemia only	Age, sex, site, year, history of incarceration, marital status, IVDU, alcohol use, homelessness, COPD, diabetes, HCV, HBV anaemia	2,217§	0.70	0.46- 1.08

Reference

1. Khan U, Huerga H, Khan AJ, Mitnick CD, Hewison C, Varaine F, et al. The endTB observational study protocol: treatment of MDR-TB with bedaquiline or delamanid containing regimens. BMC Infectious Diseases. 2019;19(1).
2. WHO. Definitions and reporting framework for tuberculosis - 2013 revision (updated December 2014). Geneva: World Health Organization; 2013. 2014.
